# The novel norovirus genotype GII.17 is the predominant strain in diarrheal patients in Shanghai, China

**DOI:** 10.1186/s13099-016-0131-3

**Published:** 2016-10-26

**Authors:** Lifeng Pan, Caoyi Xue, Huiqin Fu, Dan Liu, Linying Zhu, Chang Cui, Weiping Zhu, Yifei Fu, Sun Qiao

**Affiliations:** 1 Research Base of Key Laboratory of Surveillance and Early Warning on Infectious Disease in China CDC, Shanghai Pudong New Area Center for Disease Control and Prevention, 3039 Zhangyang Road, Shanghai, 200136 China; 2 Pudong Institute of Preventive Medicine, Fudan University, 3039 Zhangyang Road, Shanghai, 200136 China; 3 Microbe Test Section, Pudong New Area Center for Disease Control and Prevention, 3039 Zhangyang Road, Shanghai, 200136 China; 4 Department of Infectious Disease, Pudong New Area Center for Disease Control and Prevention, 3039 Zhangyang Road, Shanghai, 200136 China

## Abstract

In the winter of 2014–2015, a novel norovirus (NoV) strain (GII.17) was reported to be the major cause of gastroenteritis outbreaks in East Asia. To determine the time course of gastroenteritis infections associated with the GII.17 strain and whether GII.17 was the main epidemic strain in diarrheal patients in Shanghai, 2169 stool samples were collected and tested. The detection rate of NoV GI and GII NoV strains was 0.83 and 24.02%, respectively. Phylogenetic analysis confirmed that there were seven NoV genotypes, among which GII.4 and GII.17 were the main genotypes. The GII.17 strain was first detected in a sample collected on August 14th, 2014, and beginning in January 2015, the novel GII.17 strain replaced the GII.4 strain as the dominant NoV genotype causing acute gastroenteritis in patients in Shanghai.

Norovirus (NoV) is a leading cause of diarrhea inpatients of all age groups with acute gastroenteritis worldwide [1]. Starting in November 2014, Guangdong [2], Jiangsu [3], and Zhejiang [4] in China, and Japan [5] reported diarrheal disease outbreaks that were caused by a novel NoV genotype GII.17. Chen et al. [6] reported this novel genotype collected from sporadic patients in Shanghai during the winter of 2014–2015 has evolutionary relationships with other reported genotypes [2, 5]. Here, we characterized the molecular epidemiology of NoV infections in Shanghai between July 2014 and June 2015 to examine when did this novel NoV genotype strain begin to prevalent and whether the NoV GII.4 is still the leading NoV genotype in Shanghai, China.

A total of 2169 stool samples were collected from 18,669 diarrheal patients, reported to the syndromic surveillance network from 12 sentinel hospitals, between July 2014 and June 2015. The detection rate of the NoV GI and GII was 0.83% (18/2169) and 24.02% (521/2169) by real-time reverse transcription polymerase chain reaction (qRT-PCR), respectively.

Partial nucleotide sequences of the NoV GII ORF2 gene was amplified. Genotypes were determined using Norovirus Genotyping Tool Version 1.0. The numbers of strains corresponding to the GII.2, GII.3, GII.4, GII.6, GII.13, GII.17 and GII.21 strains were 3, 5, 133, 3, 8, 286 and 10, respectively. GII.17 (63.84%, 286/448) and GII.4 (29.69%, 133/448) were the most prevalent NoV GII genotypes between July 2014 and January 2015.

NoV infections occurred in all seasons and peaked between October 2014 and April 2015 (Fig. 1). The novel genotype GII.17 strain was first detected in a 24-year-old female patient on August 14th, 2014 in Shanghai. Beginning in January 2015, GII.17, which clustered to the novel variant Kawasaki_2014, was the most prevalent genotype of NoV GII, replacing GII.4 as the most prevalent NoV genotype in Shanghai. After May 2015, the GII.4 strain was rarely detected (Fig. 1).

**Fig. 1:**
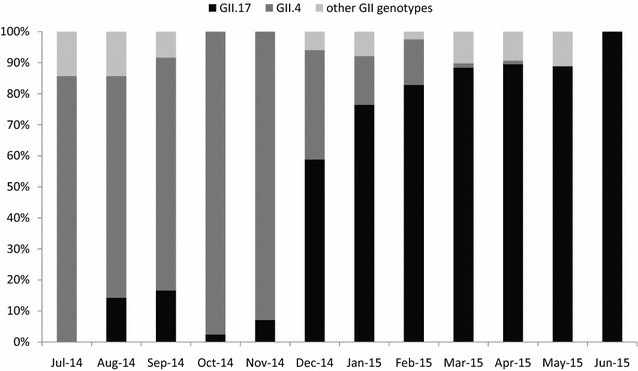
Time distribution (month) of NoV genotypes with acute diarrheal in Shanghai, China, 2014–2015

As the most prevalent strain, the GII.4 genotype Sydney_2012 and its variants were surveyed and reported globally [7–12], beginning in 2012 [13]. However, starting in November 2014, a novel NoV genotype GII.17 was reported to cause diarrheal disease outbreaks, worldwidely [2, 5, 14–16] and Matesushiam et al. [5] found that this novel genotype had a polymerase sequence and amino acid substitutions in the capsid region. In this study, we reported that the GII.17 genotype was first detected in diarrheal patients in August 2014, which was earlier than the first reported outbreak in Jiangsu [3]. Similar with the findings by Chen et al. [6], the GII.17 genotype became the predominant genotype beginning in November 2014 (Fig. 2). Our findings demonstrate that beginning in December 2014, GII.17 replaced GII.4 as the most prevalent NoV genotype in Shanghai. Teasing out viral, environmental, or host specific factors should be investigated to understand emergence of GII.17 [15] and ongoing surveillance should be performed to verify whether this novel genotype would be prevalent continuously.

**Fig. 2:**
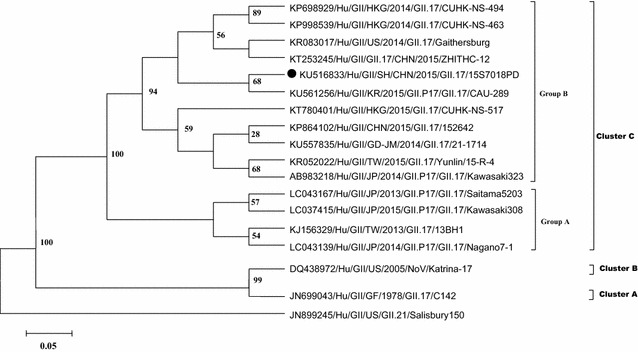
Phylogenetic analysis of partial capsid region (ORF2) of NoV strains. Presented is a cladogram with supporting bootstrap values with full length VP1 nucleotide of reference NoVs according to LeBlanc et al. [15]. Representative strain from acute diarrheal patients in Shanghai, 2014–2015 are shown with *black circle*

## Abbreviations

NoV: norovirus
SPV: sapovirus
RTV: rotavirus
ADV: adenovirus
ASTV: astrovirus
NGTV 1: Norovirus Genotyping Tool Version 1.0
qRT-PCR: real-time reverse transcription polymerase chain reaction
RT-PCR: reverse transcription polymerase chain reaction
ORF2: open reading frame 2
